# Is the Polylactic Acid Fiber in Green Compost a Risk for *Lumbricus terrestris* and *Triticum aestivum*?

**DOI:** 10.3390/polym13050703

**Published:** 2021-02-26

**Authors:** Esperanza Huerta-Lwanga, Jorge Mendoza-Vega, Oriana Ribeiro, Henny Gertsen, Piet Peters, Violette Geissen

**Affiliations:** 1 Soil Physics and Land Management Group, Wageningen University & Research, P.O. Box 47, 6700AA Wageningen, The Netherlands; oriana.ribeiro@wur.nl (O.R.); henny.gertsen@wur.nl (H.G.); piet.peters@wur.nl (P.P.); violette.geissen@wur.nl (V.G.); 2 El Colegio de la Frontera Sur, Unidad Campeche, Av. Rancho Polígono 2-A, Ciudad Industrial Lerma, Campeche C.P. 24500, Mexico; jmendoza@ecosur.mx

**Keywords:** Polylactic acid, green composts, ecotoxicology, earthworms, plants

## Abstract

Polylactic acid (PLA) bioplastic was introduced to the market as an environmentally friendly potential solution for plastic pollution. However, the effects of bioplastic debris mixed with composts on soil macroinvertebrates, plant growth and soil conditions are still unknown. Soil macroinvertebrates are soil health indicators. A reduction in their abundance is a sign of soil degradation. The objectives of this study were (i) to assess PLA debris in greenhouse composts, and (ii) to test the ecotoxicological effects of PLA debris mixed with compost on *Lumbricus terrestris*, a soil organism model, and on *Triticum aestevium,* a plant growth model. The study was comprised of three stages: (1) determine the PLA debris size distribution in composts; (2) assess the ecotoxicological effects of real-world concentrations (0% to 5%) of PLA mixed with compost on earthworm mortality and reproduction; and (3) assess the influence of compost mixed with real-world PLA concentrations on plant growth and physicochemical soil conditions. One percent of PLA debris was found in green composts, 40% of composted PLA debris measured between 1–10 mm, with a concentration of 82.8 ± 17.4 microplastics.gram^−1^ compost. A concentration of 1% PLA in composts resulted in significant mortality in earthworms. No significant effects of PLA mixed with composts were observed on plant growth or soil physicochemical conditions. Further studies are required in order to test the effect of this biopolymer on different earthworm and plant’ species.

## 1. Introduction

Among the renewable bio-based polymers, polylactic acid (PLA) is one of the most promising commercially available polymers. It is commonly derived from biomass such as vegetable fats and oils, maize starch, tapioca, or other sustainable resources [1]. Although PLA was first used for medical purposes, PLA is now used mainly for packaging and fibers [2,3] for food and non-food industries. Worldwide production of PLA had reached 180,000 tons per year by 2012 and in 2020, production was expected to rise to 800,000 tons per year [3]. PLA residues are becoming a cause of concern especially when they are not properly recycled, they are not recycled according to European EN requirements, or if ecotoxicological effects are seen such as shifts in fungal communities [3].

According to European standard EN 13432 for industrial bioplastic packaging composting and EN 14995 for industrial bioplastic non-packing composting, bioplastics must meet several conditions before they can be offered on the market. According to these standards, bioplastics must (i) disintegrate (the mass of the material residues has to become less than 10% of the original mass within three months); (ii) biodegrade (material must be converted to CO_2_ with the help of microorganisms); (iii) have no negative effects during composting (i.e., production of toxic gases); and (iv) have a heavy metal content below the maximum permissible levels (serving as soil amendment with no reduction of agronomical values or ecotoxicological effects on plant growth). The standards indicate that the industrial composting process must reach the thermophilic phase (50–60 °C) in the first weeks of composing followed by the mesophilic phase (<40 °C). Within 12 weeks, all bioplastic material has to disintegrate by 90% [4].

Although these standards have been established for PLA materials, several questions remain unanswered. Do PLA residues exist after composting? What is the concentration of PLA residues remaining in the compost after the composting process? Are these residues harmful to soil fauna or plant growth?

Greenhouse wastes have been contributing more and more PLA residues to municipal waste systems. Mainly used for packing [3], PLA fibers are commonly used in greenhouses for tomato production. The PLA fibers become fragmented and enter into the waste cycle. Although, these bio-fibers are expected to disintegrate according to the EN standards for compostable bioplastics, questions have arisen concerning PLA biodegradation. In order for PLA residues to properly biodegrade, exposure to a high temperature of 70 °C is required [4]. The common thermophilic phase in industrial composting processes only reaches temperatures between 50–60 °C.

In some European countries such as the Netherlands, the composting process is carried out in a tunnel system. Wastes are placed inside an aerobic tunnel for 2 to 3 weeks for thermophilic biodegradation at 50–60 °C (Figure S1) after which the waste is left for 5 weeks in the open air to further rot [4]. The compost is then applied to agricultural lands. At first glance, this closed cycle certainly has its benefits. However, if 100 percent of PLA degradation does not occur, the residues may create ecotoxic conditions effecting soil fauna or the physio-chemical conditions of the soils. Composts and fertilizers have been recently discovered to be vectors of microplastics [5]. Green composts are no exception since they too can carry plastic particles, although biobased, containing PLA residues. Even though several studies have to be carried out to test biobased plastics before they are launched onto the market [6], scarce information is available concerning the effects that biobased plastic residues mixed with compost may have on soil organisms, soil conditions and plants.

Plastic residues, both fossil fuel and biobased, are known as unintended plastic pollutants [7]. After environmental exposure, either to UV light and/or wind, plastic fragments breakdown into microplastics, which are particles smaller than 5 mm, and macroplastics, which are larger than 5 mm [8]. Microplastics can be moved, ingested and transported by soil fauna [9,10] which may have adverse effects on soil organisms and soil physicochemical conditions [11,12]. Forty percent of PLA residues were still found in compost after 90 days of composting under aerobic conditions [13]. There could be severe environmental implications of PLA debris being present in agricultural soils or being ingested by soil invertebrates. Various ecosystem services in which invertebrates participate could be affected by PLA residues in soil. Microplastics could also be transferred to the next trophic group and reach vertebrates [14,15] with the inevitable result that sooner or later, humans will end up ingesting microplastics (microplastic effects on humans are related to health disorders such as obesity, infertility and endocrine disruption [16]).

In this study, we wanted to answer several research questions. After the tunnel composting process followed by the opencast post-rotting process are carried out, are there still PLA residues in the green compost? What is the concentration and size of these residues? Do these residues affect soil fauna, soil conditions and plant growth?

The aims of this study were (i) to assess the concentration of macro and micro PLA debris in greenhouse composts undergoing different types of compost processes, (ii) to test the ecotoxicological effects of PLA-contaminated compost on *Lumbricus terrestris*, used as a model for soil organisms, and on *Triticaria aestevium,* used as a model for plant growth.

## 2. Materials and Methods

### Experimental Design

The study was divided into three stages (Figure S2). The aim of the first stage was to determine the concentration and size of PLA residues in composts exposed to different post-rotting times (Figure S3). The second stage focused on evaluating the ecotoxicological effects of real-world concentrations of PLAs mixed with compost on earthworm mortality and reproduction. The third stage assessed the influence of compost mixed with real-world PLA concentrations on plant growth and physicochemical soil characteristics. A list of the treatments carried out at each stage is shown in Table 1.

Stage 1. Three types of greenhouse tomato compost samples (non-composted, composted for 2 weeks in a tunnel, and composted for 2 weeks in a tunnel plus post-rotting time) with 5–10 replicates each were provided by two Dutch composting companies dedicated to the management of greenhouse wastes. The compost material was assessed as follows: The material (tomato residues plus PLA fibers) was dried in an oven at 40 °C for 3 to 4 days in order to remove any moisture content from the compost. PLA debris was then extracted and measured manually with the help of metal tweezers and a metal ruler. Microplastics (PLA debris smaller than or equal to 5 mm) were optically assessed per gram of compost with the help of a stereomicroscope, to double check that the debris was indeed PLA debris and not tomato residue. We carried out the assessment following the final step in the method of Zhang et al. [17] by taking a photo of the selected microplastic debris. A photo was taken before and after heating the sample to a temperature of 120 °C, which is the melting temperature of the composted PLA. It is important to note that the compost material used in this study was exclusively from tomato greenhouses where PLA fibers were used.

Stage 2. After being composted for two weeks in a tunnel, PLA debris in different concentrations (0, 0.1%, 0.25%, 0.5%, 0.75%, 1, 3%, 5%) was added to a commercial plastic-free compost (Pokon Naturado B.V. ^®^, Veenendaal, The Netherlands). The PLA concentrations were selected together with the stakeholder who provided the composted PLA material for the study. The size of the added PLA debris followed the dominant sizes found in step 1 of this study (Figure S1, 60–65% with 150–10 mm; 40–35% < 10 mm). The compost mixed with PLA debris was then placed over the soil surface of a 40 × 30 × 3 cm^3^ mesocosm in an amount that corresponded to 30 Tons compost/ha according to [18]. Inside the mesocosm, 2 kg of soil was added. The soil was provided by Huldenberg, Belgium, and was characterized as follows: 10% clay, 79% silt, and 11% sand, with pH of 5.8, 3.2% organic matter, 1.7 g·kg^−1^ total N, 0.4 mg·kg^−1^ available P and 39.1% *w*/*w* soil water holding capacity.

We installed 2 identical experimental setups in terrariums to simulate realistic scenarios, with 8 treatments (Table 1) each and 3 replicas per treatment. One setup was used for evaluating the effect of PLA on earthworm mortality after 14 days (adaptation of the earthworm acute toxicity test 207 [19]) and the second setup was used for determining the effect of PLA on earthworm reproduction after 60 days (adaptation of the earthworm reproduction test 222, [20]). Both experimental setups were housed in a special chamber where temperature (17 °C) and moisture (17%) conditions were controlled and constant (adequate conditions for *Lumbricus terrestris*). Even though the tests in 207 and 222 were designed for the epigeic *Eisenia fetida*, we adapted the procedures for the anecic *Lumbricus terrestris*. Until now, there have been no acute toxicity and reproduction tests for *L. terrestris*.

Four *Lumbricus terrestris* adults with an initial average weight of 4.29 ± 0.61 g were placed in each of the experimental mesocosm setups. In previous studies, following the tests, 10 earthworms were used, but due to the ecological requirements of *L. terrestris*, 4 earthworms are the adequate number inside 2 kg of soil. After either 14 days or 60 days, in accordance with the OECD tests, the terrariums were frozen and opened. Earthworm bodies and burrows were counted and the burrows were collected, counted and dried at 40 °C. Burrow weight was recorded and the volume of each of the burrows was not registered. PLA debris in burrows was extracted manually with the help of clean metal tweezers and the PLA debris concentration per burrow was determined.

At the end of each experiment, changes in individual earthworm biomass and mortality rates were recorded (body presence or body absence after freezing). Reproduction rate was determined by counting cocoon production per worm [20] and juvenile earthworms.

Stage 3. To test the effect of PLA debris mixed with compost on wheat growth and production yield, 24 18 litre pots (32 cm diameter × 37.8 cm height) were filled with loess soil from Huldenberg, Belgium (soil properties are described above in step 2). PLA debris mixed with compost at the same concentrations used in Section 2 (30 Ton/ha) was applied to the soil surface of each pot, with the same number of treatments as in Section 2 (8 treatments with 3 replicas each). Twenty-four seeds were sown at a depth of 5 cm in each pot, which was equivalent to 300 seeds per square meter. When the seeds were sown, the pots were watered to field capacity.

Pots were randomly placed in a climate-control cell and the climate parameters were set as follows: Day length of 16 h, temperature at 25 °C, and light intensity at 920 µmol·m^−2^·s^−1^, all of which correspond to the European Summertime. Pots were watered every day and soil moisture was monitored with TDR equipment to achieve 16–23% (*w*/*w*). A nutrient solution with macro and micronutrients was also supplied regularly to the plants to support optimal crop growth.

Soil samples from the pot experiments were collected and the following parameters were determined: Soil bulk density by the core method [21], soil aggregate stability by wet-sieving [22], water permeability by minidisk infiltrometer [23], and N mineralization using 1M KCl solution [24] according to the OECD guideline for soil chemical testing [25].

Statistical analyses: In order to determine whether there were statistically significant differences among the treatments for all studied parameters, several analyses were carried out. After verifying the homogeneity of the data and if the data followed a normal distribution, one-way ANOVA followed by post-hoc Tukey and Dunnett’s analysis (for exploring all possible pairwise comparisons among groups and treatments-to-control comparisons) as well as linear model analysis were performed. For data without normal distribution, a non-parametrical analysis was carried out (Kruskal–Wallis, followed by Mann–Whitney U Test). Furthermore, Principal component analysis based in correlation matrices, a technique for finding patterns in data of high dimension described by Meng amd Yang [26], was performed with CANOCO software in order to observe how the response variables were correlated and disposed on the factorial plan together with the treatments.

## 3. Results

### 3.1. PLA Residues in Composts Exposed to Different Types of Composting Treatments

The size and concentration of PLA debris in green composts were dependent on the type of composting. We observed how PLA fiber residues degraded under compost conditions. PLA debris concentration in the composts was 10 times lower than in the non-composted material (0.82 ± 0.11 % to 1 ± 0.51 % w.w, Table S1). No significant difference was observed between the concentration of PLA in composts processed only in the tunnel versus the composts processed in tunnels and then exposed to outdoor post-rotting (Table S1). Forty percent of the composted PLA debris fell within a size range of 1 to 10 mm (Figure S4), with an average concentration of 82.8 ± 17.4 microplastics·gram^−1^ compost (Figure S5).

### 3.2. Ecotoxicological Effects of PLA Residues in Composts

#### 3.2.1. Ecotoxicological Effects of PLA Residues on Earthworms

Although significant effects on earthworm mortality and growth were expected, earthworm mortality was observed only at the following PLA concentrations: 0, 0.75% and 1% (Table 2). However, due to the absence of statistical differences, no significant differences among treatments were found and the biomass change (Table 2) was not significantly different among treatments. No reproduction took place in any treatment during the experiment. Indeed, it is difficult to explain why mortality was also present in the control and not in treatments with higher concentrations of PLA. There could be two possible explanations for this. The first is that the earthworm set used was not healthy from the start thus, researchers should look into reproducing their own earthworms for experiments. The second explanation could be that the earthworms did not die when exposed to high concentrations of PLA because of avoidance behavior. The earthworms simply did not ingest the material when it was present in high concentrations.

#### 3.2.2. Ecotoxicological Effects of PLA on Plant Growth

Plant growth was not affected significantly in any of the treatments. There were no significant differences among treatments in relation to growth and seed production. The highest mean yield (11.3 ± 0.44 Tonnes.ha^−1^) was observed in the treatment with the lowest concentration of microplastics in the compost (0.1%, Table 3).

#### 3.2.3. Effects of PLA on Soil Physicochemical Conditions

No significant effects of the treatments were observed on the soil physical and chemical parameters measured in this study. Aggregate stability, unsaturated hydraulic conductivity, bulk density and nitrogen mineralization rate (%) did not seem to be affected by composted PLA, even though treatments with low PLA concentrations in the compost had lower aggregate stability (52.8–53.3%) than treatments with higher concentrations of PLA (64.5 %and 71.25%). The highest, but not significant, mineralization rate was found in the control treatment (Table 4).

#### 3.2.4. Effects of PLA on Earthworm Burrows

**Figure 1:**
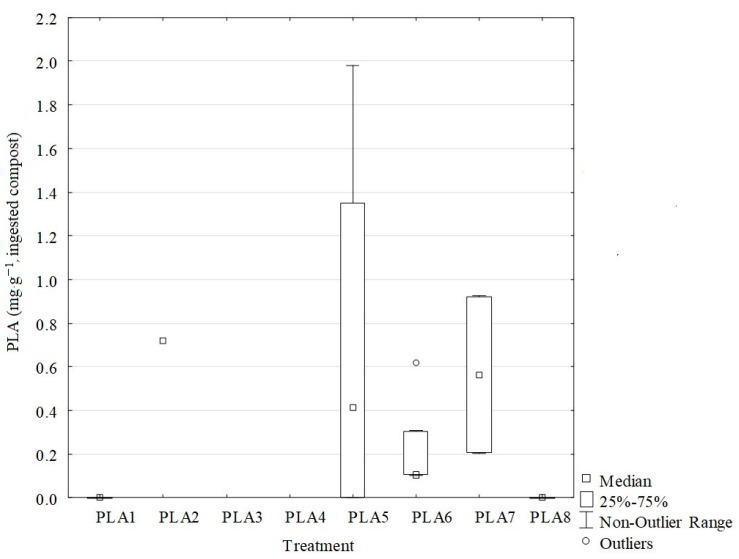
Concentration of polylactic acid (PLA) in earthworms burrows (ingested compost, mg·g^−1^).

PLA transport via earthworms was witnessed by the PLA content found in the tunnels of *Lumbricus terrestris*. PLA debris in burrows was only present in treatments with 0.25%, 1%, 3% and 5% PLA (mg·g^−1^ of PLA per ingested soil, Figure 1). The concentration of PLA debris per gram of ingested soil was higher in the 1% PLA treatment, but only significantly different in the 0.25% PLA treatment (Figure 1). The weight of the burrows was not significantly different between the treatments (9.6 ± 0.04 g dried weight). The number of burrows in the 1% PLA treatment was higher, but not significantly different.

#### 3.2.5. Relationship between All Response Variables

**Figure 2:**
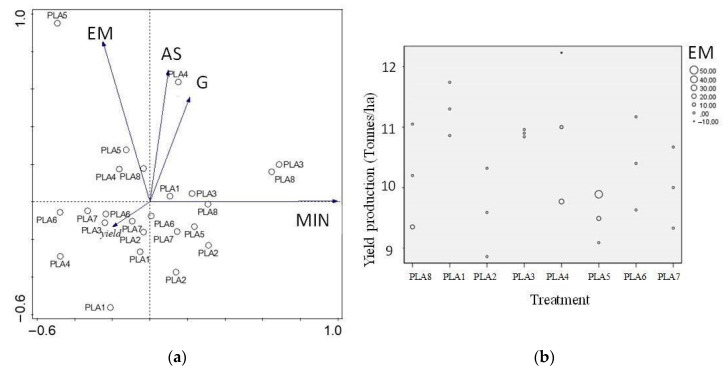
(**a**)Variables and treatments under the factorial plan, after a principal component analysis (PLA 1–8, 1: 0.1%, 2: 0.25%, 3: 0.5%, 4: 0.75%, 5: 1%, 6: 3%, 7: 5 %, 8:0%); EM: Earthworm mortality, AS: Aggregate stability, MIN: Nitrogen mineralization rate; (**b**) Plant yield production and earthworm mortality rate (EM) under PLA with compost.

Using principal component analysis (PCA) helped to situate the different response variables in relation to each other and the treatments. Even though the variables belong to different groups (plant, earthworms and soil responses), they were analyzed together as response variables to the treatments. This principal component analysis showed correlations between soil aggregate stability and grain production, which are found on the same axis with 96.6% of variation explained on the factorial plan (Figure 2a). These values belong to the 0.75% PLA treatment, which enhanced that behavior. These two variables were completely opposite of yield production, nitrogen mineralization rate and earthworm mortality. There was no correlation among these three variables and the soil aggregate stability and the production of grains in this study.

Even though earthworm mortality and yield production had no correlation (Figure 2a), the highest earthworm mortality was seen in the pot with the lowest yield production (1% PLA treatment) in this study (Figure 2a,b). Even though the linear model analysis showed a significant effect on earthworm mortality (*p:* 0.01) under 1% PLA, mortality was also observed in the control treatment, so we need to be very careful with this assumption.

## 4. Discussion

The composting process is a very noble and economical process in which organic waste is processed and recycled. The product is a nutrient-rich amendment which is beneficial for soil life and plants. Green composts are the result of managing the green waste collected from parks, gardens, domestic dwellings [27], and green houses. The exponential increase of green compost is accompanied by the exponential growth of cities. Unfortunately, green composts often have low levels of micro-pollutants [27], which is a big concern. Microplastics found in green compost are small (<5 mm) pieces of biodegradable plastics which originate from the use of biodegradable packaging or fibers.

These biodegradable wastes should normally decompose during the composting process and be incorporated into the recycling chain. However, if the composting process does not allow the bioplastic residues to degrade completely then the bioplastic residues become macro and microplastic pollutants.

Polylactic acid (PLA) bioplastic is one of the main plant-based plastics used worldwide. In our study, we confirmed that after tunnel composting, either with or without exposure to the outdoor post-rotting process, the maximal concentration of PLA fiber residues in compost is 1%. According to our results, this concentration is not harmful to *T. aestevium* growth, *L. terrestris* mortality or soil characteristics.

Due to the high variability and the low number of repetitions per treatment in this study, it was difficult to discern the treatment effects. The principal component analysis showed that concentrations of 1% and 0.75% PLA were significant enough to influence earthworm mortality. However, mortality was also observed in the control treatment with 0% PLA which means that even though the mortality mean in these 3 treatments was similar, in treatment with 1% PLA, more worms died per terrarium than in the other 2 treatments. These findings suggest that more repetitions are required in order to obtain stronger effects to allow researchers to better discern among the treatments. Previous studies have shown how biobased microplastics may influence plant growth or soil conditions [11,28], which was not the case in this study.

The certified and compostable polylactic acid biobased plastic (PLA) derived from renewable resources, such as plant sugars [1], is widely used to make fibers needed for tomato crops in greenhouses, and in plastic mulches [2]. It holds first place in the market among biobased plastics, and it is the most well-established new biobased polymer. Although our study showed not effects of this polymer, more studies are recommended to test this material on different earthworm species for longer periods of time with >3 repetitions per treatment in order to test PLA short-term and long-term effects under different environmental conditions and with different soil types.

Reports related to PLA debris from mulches have shown that this material does not degrade inside the bulk soil [2,7] unless high temperatures are reached. This seems to occur with PLA residues from green composts since 40% of the PLA residues were still found in composts after 90 days of composting when high temperatures (70 °C) were not reached even with aerobic digestion [29]. In our study, 0.8 to 1% of PLA residues were found in the compost after the tunnel process, indicating that the temperature present in the tunnel (70 °C) plays an important role on the degradation or lack of degradation of this bioplastic. We verified with the Zhang et al. [17] method that this composted material could be melted at 120 °C.

Still, if 1% of the PLA residues remain in compost and the compost is applied, questions in relation to the effect of this material on different environmental conditions and soil types arise. In our study, under temperate conditions (17–23 °C) and with silt loam soil, no effects were observed. Further studies are required in order to identify potential effects of PLA residues under different climatological conditions and for different soil types.

In our study, we only observed that PLA fiber debris, when mixed with compost with a concentration higher than 1% but lower than 5%, can be ingested and transported by soil invertebrates. What kind of PLA decomposition process may take place inside the earthworms or earthworm burrows it is still unknown. The purpose of our study was to confirm that in certain concentrations, earthworms can ingest PLA residues and can transport them into deeper layers, seemly causing no significant harm to the soil conditions or to the plants. Reference [29] already established that PLA mulch degradation depends highly on the type of soils where it is present.

Fossil fuel-based microplastics and some biobased microplastics might influence plants-soil systems which has been observed with *Allium sepa* [30] and *T. aestivum* [28]. Modified plant growth was not witnessed in our study. PLA microplastic fibers did not seem to influence *T. aestivum*, neither plant growth nor seed production were affected. It is important to mention that previous experiments were developed to be performed for 2 to 4 months, and our plant experiment was performed in 2 months. A longer-term experiment should be conducted in order to observe possible longer-term effects of PLA fiber debris on plants. Our study confirmed that PLA bioplastic had no observed effect on plant growth or seed production during a 2 month experiment.

## 5. Conclusions

PLA debris was ingested by *L. terrestris* and only when the concentration of this material was equal to or higher than 1% was it found in earthworm burrows. Plant growth and seed production was not affected by PLA debris mixed with composts. Soil chemical and physical parameters measured in this study were not significantly affected by the treatments.

## Figures and Tables

**Table 1:** Experimental design and treatment description.

Stage	Treatment	Description	Assessment
1	2 wkT	2 wk tunnel (70 °C)	Green compost
1	2 wk T + n	2 wk tunnel+O	Green compost
2 and 3	PLA1	0.1 % PLA	Earthworms, plants, soil
2 and 3	PLA2	0.25 % PLA	Earthworms, plants, soil
2 and 3	PLA3	0.50 % PLA	Earthworms, plants, soil
2 and 3	PLA4	0.75 % PLA	Earthworms, plants, soil
2 and 3	PLA5	1 % PLA	Earthworms, plants, soil
2 and 3	PLA6	3 % PLA	Earthworms, plants, soil
2 and 3	PLA7	5 % PLA	Earthworms, plants, soil
2 and 3	PLA8	0 % PLA	Earthworms, plants, soil

wk: Weeks, T:tunnel, O: Outdoor compost (7–12 °C), n: Number of weeks outdoors (4–13).

**Table 2:** Earthworm biomass change (% change of initial biomass/earthworm) and mortality after two weeks of exposure to pla. (mean, sd). No significant differences among treatments according to one way anova, tukey pairwise comparison and dunnett’s treatments-to-control comparisons.

Treatment	Biomass Change (%) Means (SD)	Mortality (%) Means (SD)
PLA1	−17.4 (6.35)	0 (0)
PLA 2	−11.9 (3.19)	0 (0)
PLA 3	−15.4 (10.0)	0 (0)
PLA 4	−3.87 (8.42)	8.33 (14.4)
PLA 5	−2.36 (12.1)	16.7 (28.9)
PLA 6	−14.8 (6.86)	0 (0)
PLA 7	−11.6 (7.68)	0 (0)
PLA 8	−14.3 (12.2)	16.7 (14.4)

**Table 3:** Comparison of wheat growth parameters among treatments. Mean values and (Stdev). No significant differences were found among treatments according to one way ANOVA.

Treatment	Parameters					
	Number Grain/Ear	Weight/Grain (g) (n.s.)	Weight Grain/Ear (g)	Number of Tillers (m^2^)	Plant Dry Matter (g/m^2^)	Yield Grain (t/ha)
PLA1	34.2 (3.71)	0.04 (0.001)	1.33 (0.14)	1004 (67.7)	1091 (129)	11.3 (0.44)
PLA 2	31.3 (0.87)	0.04 (0.001)	1.15 (8.05)	1023 (35.6)	1039 (128)	9.59 (0.73)
PLA3	35.5 (1.93)	0.04 (0.001)	1.36 (0.06)	940 (44.3)	1030 (34.1)	10.9 (0.06)
PLA 4	33.0 (2.02)	0.04 (0.001)	1.35 (0.11)	950 (32.9)	1161 (87.3)	11.0 (1.23)
PLA 5	33.6 (3.10)	0.04 (0.002)	1.22 (0.05)	917 (11.9)	1017 (56.4)	9.49 (0.40)
PLA 6	34.4 (0.63)	0.04 (0.002)	1.35 (0.11)	904 (47.1)	1069 (127)	10.4 (0.77)
PLA 7	34.4 (2.12)	0.04 (0.001)	1.30 (0.09)	910 (57.2)	1002 (84.3)	10.0 (0.67)
PLA 8	34.9 (3.32)	0.04 (0.001)	1.29 (0.13)	928 (25.1)	1065 (63.5)	10.2 (0.85)

**Table 4:** Comparison of soil parameters among treatments. Mean values (Stdev). No significant differences among treatments according to one way ANOVA and Tukey pairwise comparison. * No significant differences among treatments according Kruskal–Wallis non-parametric analysis.

Treatment	Parameters					
	Aggregates >250 µm (%)	Unsaturated * Hydraulic Conductivity (cm h^−1^)	Bulk Density (g cm^−3^)	Time Cero: N-(NO_3_+NO_2_) (mg/kg soil)	28 days Incubation: N-(NO_3_+NO_2_) (mg/kg soil)	Rate of Mineralization N (%)
PLA1	53.3 (16.9)	0.43 (0.03)	1.12 (0.04)	19.9 (1.76)	65.5 (3.46)	231 (41.3)
PLA 2	52.8 (7.42)	0.16 (0.07)	1.17 (0.02)	20.0 (2.80)	75.5 (1.00)	281 (45.3)
PLA 3	70.5 (5.37)	0.12 (0.05)	1.12 (0.03)	19.8 (3.08)	77.7 (16.0)	303 (121.0)
PLA 4	71.2 (6.39)	0.19 (0.05)	1.15 (0.05)	24.2 (9.87)	69.2 (16.2)	202 (82.4)
PLA 5	65.5 (5.47)	0.12 (0.03)	1.13 (0.03)	22.8 (3.31)	69.3 (11.8)	212 (94.5)
PLA 6	67.8 (2.72)	0.10 (0.14)	1.12 (0.04)	27.3 (3.18)	76.7 (13.3)	183 (63.4)
PLA 7	64.09 (5.31)	0.16 (0.10)	1.07 (0.08)	21.00 (3.13)	66.0 (3.28)	220 (62.3)
PLA 8	66.15 (7.48)	0.15 (0.06)	1.15 (0.02)	20.0 (3.90)	83.3 (13.8)	325 (88.7)

## Data Availability

Data from soil, plants and earthworms are available if required.

## Supplementary Materials

The following are available online at https://www.mdpi.com/2073-4360/13/5/703/s1. Figure S1. Industrial green compost process in the Netherlands; Figure S2. Research stages. PLA in compost assessment and ecotoxicological effects on earthworms and plants. Figure S3. PLA% determination procedure in composts; Figure S4. PLA debris (macro, meso and micro) size distribution; Figure S5. PLA microplastics (particles.g-1 compost); Table S1. PLA % (w.w) per green compost exposed to different composting methods and composting durations.
