# Supplementary material for: Is the Polylactic Acid Fiber in Green Compost a Risk for Lumbricus terrestris and Triticum aestivum?
Source: Polymers (Basel). 2021 Feb 26;13(5):703. doi: 10.3390/polym13050703 (PMC7956588; doi:10.3390/polym13050703)
Supplement: Supplementary file 1 [file polymers-13-00703-s001.pdf]

Supplementary Materials:

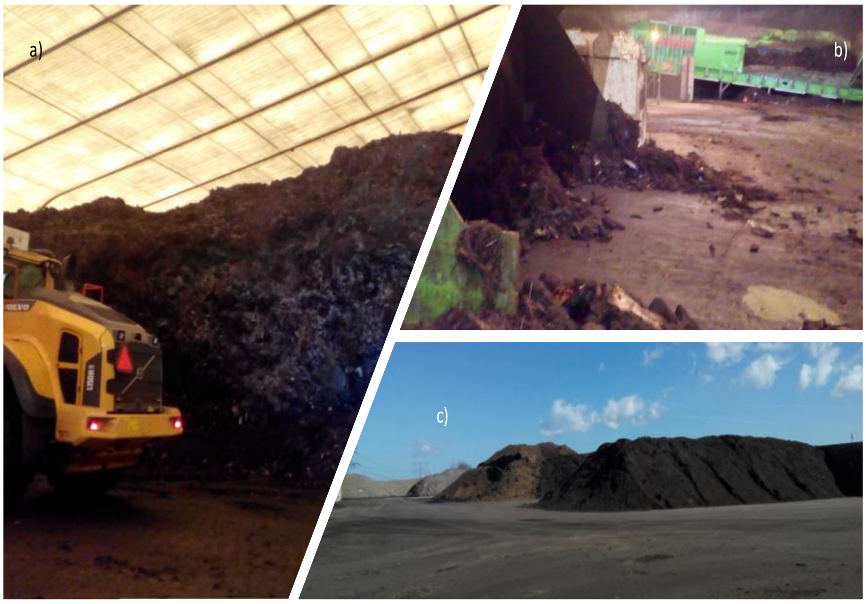

Figure S1. Industrial green compost process in the Netherlands; left : organic wastes pile; upper right: organic wastes kept for 2 weeks inside a tunnel (where 70ºC is reached); lower right: outdoor organic waste management, wastes are exposed to open air, to further the post-rotting process.

PLA in compost assessment

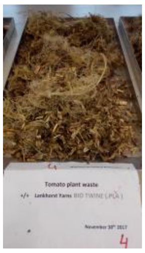

Assessment	Endpoint
After tunnel & tunnel + outside	PLA size distribution (Macro & Micro)
After tunnel & tunnel + outside	PLA Macroplastics concentration (w/w)
After tunnel & tunnel + outside	PLA microplastics concentration (w/w)

Ecotoxicological assessment

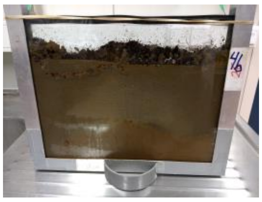

Assessment time (days)	Endpoint
60	Mortality
14	No. Burrows
14	PLA transportation in burrows
60	Growth
60	Reproduction

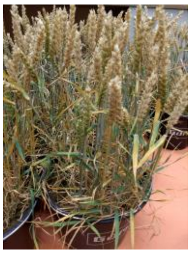

Assessment time (days)	Endpoint
60	Mortality
60	Grain number per ear
60	Growth
60	Yield production

Figure S2. Research stages. PLA in compost assessment and ecotoxicological effects on earthworms and plants. Soil condition characterization under PLA and compost were determined in the plant experiment.

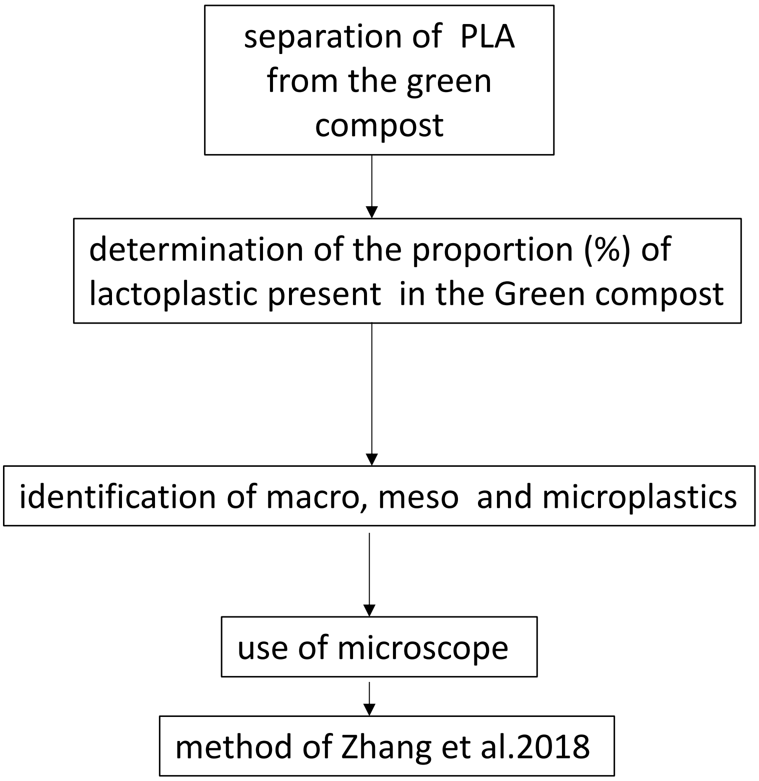

Figure S3. PLA% determination procedure in composts.

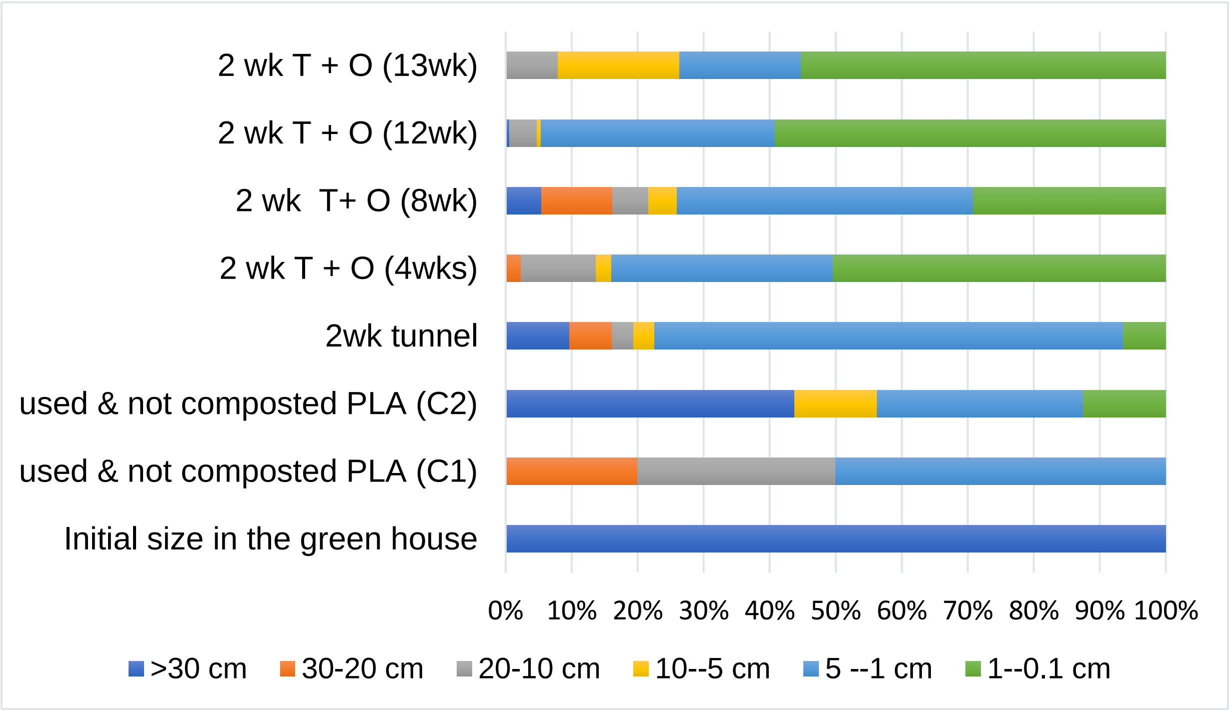

Figure S4. PLA debris (macro, meso and micro) size distribution. Wk: weeks, T: tunnel process, O: outdoor process. C1 & C2: companies 1 and 2 respectively.

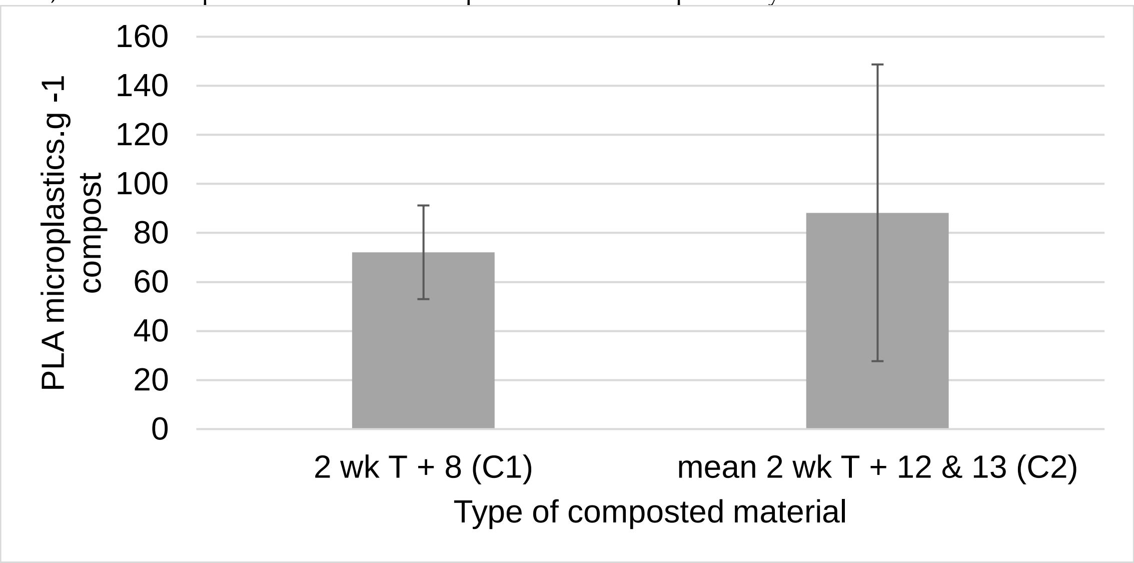

Figure S5. PLA microplastics (particles.g-[1] compost). Wk: weeks, T: tunnel process. C1 & C2 companies 1 and 2 respectively.

Table S1. PLA % (w.w) per green compost exposed to different composting methods and composting durations. NC: not composted, Wk: weeks, T: tunnel method with aeration at 70ºC, O: outdoor composting also termed post-rotting process.

		**Compost**	**Composting weeks**		**PLA % (w.w) per compost**			
**Treatment**	**N**	**method**	**T**	**O**	**Mean**	**Minimum**	**Maximum**	**Std. Dev.**
NC (C1)	8	NC	0	0	43.93	31.18	63.16	12.23
NC (C2)	2	NC	0	0	33.26	30.19	36.33	4.34
Mean (C1 & C2)	10	NC	0	0	41.79	30.19	63.16	11.78
Mean 2wk T(C1)	5	T	2	0	0.82	0.71	1	0.11
2 wk T + 4 (C2)	6	T + O	2	4	1.04	0.67	1.58	0.39
2 wk T + 8 (C1)	2	T + O	2	8	1.61	1.18	2.04	0.61
2 wk T + 12 (C1)	1	T + O	2	12	1.03	1.03	1.03	*
2 wk bunker + 13 (C2)	1	T + O	2	13	0.26	0.26	0.26	*
Mean 2 wk T + O	10	T + O	2	4, 8, 12, 13	1.08	0.26	2.04	0.51
